# Species delimitation in the cyanolichen genus *Rostania*

**DOI:** 10.1186/s12862-020-01681-w

**Published:** 2020-09-10

**Authors:** Alica Košuthová, Johannes Bergsten, Martin Westberg, Mats Wedin

**Affiliations:** 1 Department of Botany, Swedish Museum of Natural History, P.O. Box 50007, SE-104 05 Stockholm, Sweden; 2 Department of Zoology, Swedish Museum of Natural History, P.O. Box 50007, SE-104 05 Stockholm, Sweden; 3 Museum of Evolution, Uppsala University, Norbyvägen 16, SE-752 36 Uppsala, Sweden

**Keywords:** Biodiversity, Fungi, Integrative taxonomy, Lichens, Phylogeny, Symbiosis, Systematics

## Abstract

**Background:**

In this study, we investigate species limits in the cyanobacterial lichen genus *Rostania* (*Collemataceae, Peltigerales, Lecanoromycetes*). Four molecular markers (mtSSU rDNA, β-tubulin, MCM7, RPB2) were sequenced and analysed with two coalescent-based species delimitation methods: the Generalized Mixed Yule Coalescent model (GMYC) and a Bayesian species delimitation method (BPP) using a multispecies coalescence model (MSC), the latter with or without an a priori defined guide tree.

**Results:**

Species delimitation analyses indicate the presence of eight strongly supported candidate species. Conclusive correlation between morphological/ecological characters and genetic delimitation could be found for six of these. Of the two additional candidate species, one is represented by a single sterile specimen and the other currently lacks morphological or ecological supporting evidence.

**Conclusions:**

We conclude that *Rostania* includes a minimum of six species: *R. ceranisca*, *R. multipunctata*, *R. occultata* 1, *R. occultata* 2, *R. occultata* 3, and *R. occultata* 4,5,6. Three distinct *Nostoc* morphotypes occur in *Rostania*, and there is substantial correlation between these morphotypes and *Rostania* thallus morphology.

## Background

Species delimitation as a topic has experienced a renaissance since the turn of the millennium [1]. Largely driven by the increasing use of molecular data in both phylogenetics and population genetics, the two historically parallel research fields now contribute different methodological and conceptual aspects to the interface where they meet in species delimitation and population divergence research [2]. However, while the genomic era offers ever increasing power to detect small differences between populations it is in no means the magic bullet to the problem of species delimitation [3, 4]. As an illustration, even with genomic data at hand, researchers still disagree about how many species of giraffes, orangutans or Darwin ground-finches exist [5, 6]. Recent appeals discourage relying on genomic data alone for species delimitation and plea for an integrative approach including information on phenotypic and ecological variation [3, 6], in essence the concept of integrative taxonomy [7, 8].

Although not solving all problems with species delimitation, new molecular technologies have boosted the discovery of undescribed diversity in the lichen symbiosis. Until relatively recently, lichens have been viewed as a symbiosis between a single fungus (the mycobiont) and one or two photosynthesizing symbionts (the photobionts) which the fungus house within a structure called the lichen thallus. Now, we know that lichen thalli also host diverse communities of non-photosynthetic bacteria (e.g. [9–12]) but these seem not to affect the morphology of the thallus as far as we currently know. Additional fungal elements, known as lichenicolous fungi, may influence thallus morphology [13] through the formation of galls. Common basidiomycete yeasts have been suggested to influence thallus morphology to some extent, but their role in the symbiosis is still hypothetical [14–16]. It has been known for quite some time that one mycobiont may form very dissimilar lichen thalli with very different photobionts [17–19]. While it is also clear from several studies that lichen fungi frequently recruit several distinct lineages of photobionts [20–24], the impact of different strains of a photobiont genus on the resulting thallus morphology is much less understood.

**Fig. 1:**
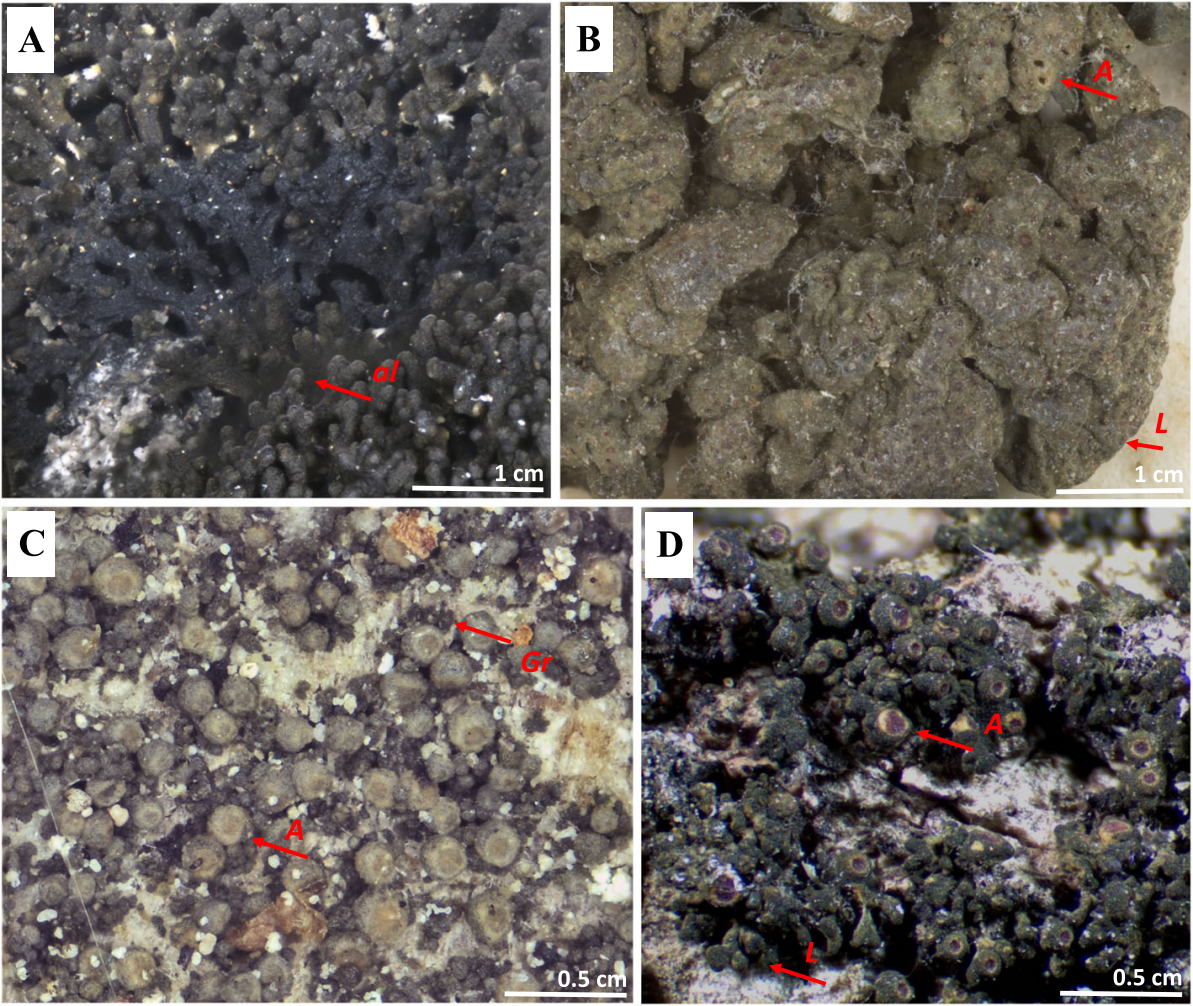
Traditionally recognised taxa based on Degelius’ morphological hypotheses from 1954 [19]. Thallus habitus and the colour of the apothecia. **a** *Collema ceraniscum* (S-F262465) –accessory lobules on the top of the lobes (arrow), **b** *C. multipunctatum* (Nyl41670_H9201930) – lobate thallus with large lobes (arrow), **c** *C. occultatum var. occultatum* (S-F388771) – granulose thallus (arrow) and pale brown coloured disc of the apothecia (arrow), **d** *C. occultatum var. populinum* (UPS-L125813) – lobate thallus composed of small lobes (arrow) and red coloured disc of the apothecia (arrow), ***al***–accessory lobule, ***A***–apothecium, ***Gr***–granule, ***L***–lobe

In our continuing studies on the systematics of the cyanobacterial “jelly lichens” in the *Collemataceae* [25–27] we here investigated the species delimitations in the recently re-established genus *Rostania* [26]. *Rostania s. str.* corresponds to the informal *Collema* “Occultatum” group of Degelius (1954) [28], which included small species with poorly developed lobes, small apothecia and cubic or similar spores. In this group Degelius originally placed four European taxa; *Collema ceraniscum* (Fig. 1a), *C. multipunctatum* (Fig. 1b)*, C. occultatum var. occultatum* (Fig. 1c), and *C. occultatum var. populinum* (Fig. 1d). Degelius distinguished these taxa on morphological grounds, where *C. ceraniscum* differed from the others in its terricolous habit, a lobate thallus with accessory teretiform (finger-like) lobules and oval spores. *Collema multipunctatum*, a more southern species, was distinguished primarily by its large and more distinctly lobate thallus. The two varieties of *C. occultatum* (both epiphytic and with cubic spores) were rather vaguely delimited, where var. *populinum* had a coarser, somewhat lobate thallus and larger apothecia than the crustose, granulose var. *occultatum*, and was found in the south of Scandinavia completely restricted to *Populus* as a substrate. In our earlier study [29], *Rostania* was shown to be monophyletic, when only three of the currently accepted species (*R. ceranisca*, *R. multipunctata* and *R. occultata*) were included in *Rostania s. str.* In this restricted sense *Rostania* was characterized by a crustose to subfoliose thallus, with initially immersed apothecia which only become sessile late in the development, and with a disc that is concave when young and never convex. The spores were muriform with at least five cells, cuboid to oblong in shape, but never fusiform. Most species were small in comparison with other *Collemataceae*, and all lack cortex, rhizines and isidia.

Our study [29] further showed that *R. occultata* is not monophyletic in the current circumscription. To some extent, this was already recognised through the two more or less distinct varieties suggested to occur in this species [28, 30]. Further preliminary studies of the morphological and anatomical variation in *Rostania occultata s. lat.* suggested that there was substantial general variation in thallus morphology supporting multiple morphologically distinct but previously unrecognized species, which spurred an extended molecular study of the species delimitations in the group that we will present here. We further observed a surprising variation in photobiont appearance in *R. occultata s. lat*. The literature suggests that the *Nostoc* occurring symbiotically in *R. occultata* form chains of cells [28, 31]. We could however observe at least three morphologically distinct *Nostoc* types in our material; one forming small clusters of cells, one forming short chains of cells and one forming long chains. This led us to consider if the *Rostania* species also are characterized by their cyanobacterial photobiont specificity.

The biodiversity in many, if not most, fungal groups is surprisingly poorly known. Many cyanolichen groups related to *Rostania* have recently been the subject of major studies, where substantial amounts of overlooked biodiversity has been recovered. Examples include large foliose lichen groups such as *Pseudocyphellaria* [32, 33] *Sticta* [34] and *Peltigera* [35] but also more closely related groups in the Collemataceae like *Leptogium* [36]. Considering the substantial morphological variation and the indications from our earlier phylogenetic studies, *Rostania* is likely to contain several yet unrecognised species, even in a well-documented area like Europe. The current species delimitations in *Rostania*, which are based on Degelius’ morphological hypotheses from 1954 [28], still requires testing in a modern phylogenetic context.

Coalescent-based species delimitation methods using molecular data are increasingly used as part of the integrative taxonomical toolbox [37]. Together with morphology, geography, ecology and other data sources, coalescent-based species delimitation methods becomes part of the evidence to test species limits in a unified species concept framework [37]. We used two such methods for this study and apply de Quieroz’s unified species concept [38] that largely reconciles the diversity of contemporary species concepts. Under the unified species concept (or general lineage concept [39]), species are separately evolving metapopulation lineages. Multiple sources of evidence can be used to infer separate evolution of lineages, such as reproductive isolation, diagnostic morphological characters or reciprocal monophyly in gene trees, and these characteristics appear at different times during the speciation process. The unified species concept is elegantly suited to partner with an integrative taxonomic framework [37]. In empirical species delimitation inquiries, rejecting a one-species null model should preferably be supported by at least two different indications of separate evolution of lineages, often coming from separate data sources [38]. For coalescent-based species delimitation we used the General Mixed Yule Coalescence model (GMYC) [40, 41] to evaluate species limit information from each gene individually, and the Bayesian method BPP for multilocus species delimitation based on the multispecies coalescent model [42, 43]. Both GMYC and BPP have been applied to many animal and plant groups (e.g., [44–50]) as well as in studies of fungi including lichens (e.g. [34, 51–56]).

Here, we will test alternative species delimitations in *Rostania s. str.* with coalescent-based species delimitation methods on a new multi-locus dataset utilizing recently collected material and herbarium samples. We will also investigate if the presence of the three *Nostoc* morphotypes observed correlate with any *Rostania* morphology or with the phylogenetic species as delimited in this study.

## Results

### Taxon sampling

We produced 218 new sequences (Additional file 1) (53 mtSSU rDNA, 37 β-tub, 26 MCM7, 42 RPB2 5–7 and 34 RPB2 7–11) and added 26 relevant published sequences [26, 29]. The concatenated matrix included 63 terminals. In total, there were 4032 nucleotide positions (825 bp for mtSSU, 624 bp for β-tub, 621 bp for MCM7, 1962 bp for RPB2 5–11) out of which 785 were parsimony informative (66 bp for mtSSU, 127 bp for β-tub, 167 bp for MCM7, 425 bp for RPB2 5–11). We performed species delimitation with GMYC on each gene separately (63 sequences for mtSSU, 46 sequences for β-tub, 33 sequences for MCM7, 47 sequences for RPB2 5–11). For the BPP analyses we used the full dataset for analyses based on an 8 species guide tree (see material and methods). For a second set of BPP analyses with a 13 species guide tree derived from the β-tub GMYC results, we produced a reduced dataset including only terminals that could be assigned to the guide tree (i.e. excluding 17 terminals lacking β-tub). This reduced dataset included 46 terminals, had the same concatenated alignment length as the full dataset (4032 bp), out of which 750 were parsimony informative characters (64 bp for mtSSU, 128 bp for β-tub, 157 bp for MCM7, 401 bp for RPB2 5–11).

### Species delimitation analyses GMYC

**Fig. 2:**
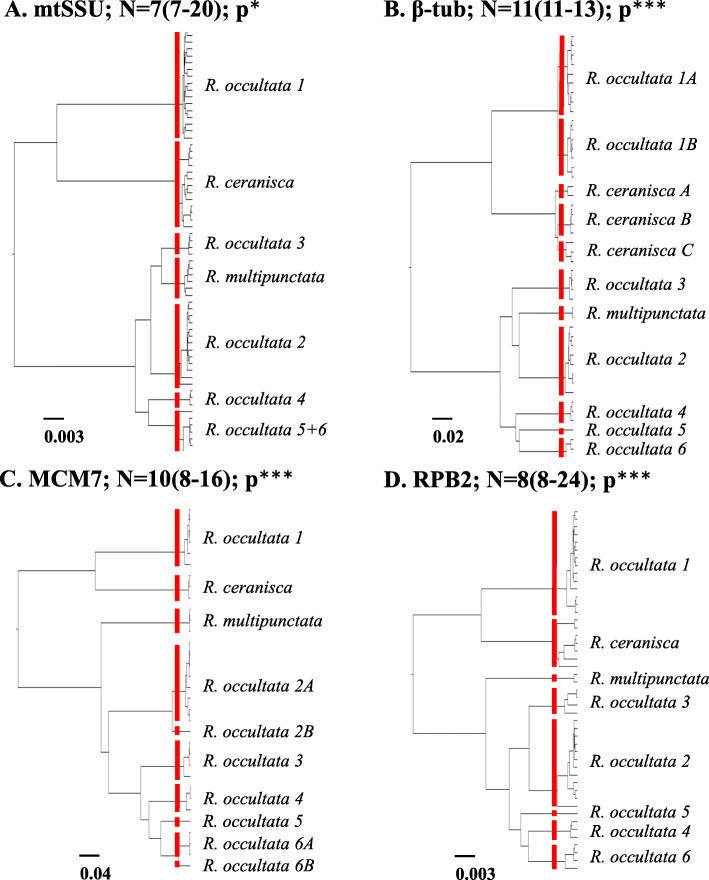
Single locus species delimitation analyses with the GMYC model. The red lines represent the maximum likelihood solution for a single threshold between interspecific and intraspecific branching. **a** mtSSU, **b** β-tub, **c** MCM7, **d** RPB2. N indicates number of delimited species at ML solution (confidence interval). Asterisks indicate significance of the likelihood ratio (LR) test (*p-value < 0.05, **p-value < 0.01 and ***p-value < 0.001- highly significant). The scale bars indicate expected number of substitutions per site

The GMYC analyses delimited between seven to eleven species at the maximum likelihood solution when the four loci were analysed individually (Fig. 2 a-d). For all genes, the log likelihood of the GMYC model was significantly better than the null model of a single coalescence in the likelihood ratio test (*p* < 0.05, Additional file 2). Notably, the two log likelihood confidence intervals never included fewer than seven species and seven species was only delimited with the mitochondrial marker. All three nuclear genes additionally delimited a single specimen from Greece (*R. occultata* 5, AL264) as a separate species (Fig. 2 b-d) but this specimen was part of *R. occultata* 6 in the GMYC result of the mitochondrial gene (Fig. 2a). This was the only difference between mtSSU and RPB2 apart from topological rearrangements (Fig. 2 a, d). MCM7 delimited 10 species and this involved a subdivision of both *R. occultata* 2 (2A and 2B-singleton) and 6 (6A and 6B-singleton) (Fig. 2c). β-tub delimited 11 species but this did not involve a subdivision of *R. occultata* 2 and *R. occultata* 6 but instead a subdivision of *R. occultata* 1 (1A and 1B) as well as three separate clades of *R. ceranisca* (A, B and C) (Fig. 2b). These latter subdivisions however are clearly the least divergent of the delimited species by β-tub as seen from the branch length estimates by BEAST (Fig. 2b).

### Bayesian species delimitation analyses BPP

The guided and unguided settings with an 8 or 13 species analyses, and each with four different prior combinations, gave sixteen BPP analyses, each run twice (Table 1). The two run replications converged satisfactory to very similar results in all cases although with slightly larger variance in the 13 species analyses. Using algorithm 0 or 1 [43], estimating theta or integrating it out [42] had no significant effect on any of our main conclussions (Additional file 3 A-D).

**Table 1:** BPP analysis strategy. Value in parenthesis for the priors is the mean of the inverse gamma distribution

Maximum number of species:	8		13	
Guide tree:	guided	unguided	guided	unguided
Prior combinations				
tau(0.1), theta(0.1)	×2 runs	×2 runs	× 2 runs	× 2 runs
tau(0.1), theta(0.01)	×2 runs	×2 runs	× 2 runs	× 2 runs
tau(0.01), theta(0.1)	×2 runs	×2 runs	× 2 runs	× 2 runs
tau(0.01), theta(0.01)	×2 runs	×2 runs	× 2 runs	× 2 runs

**Fig. 3:**
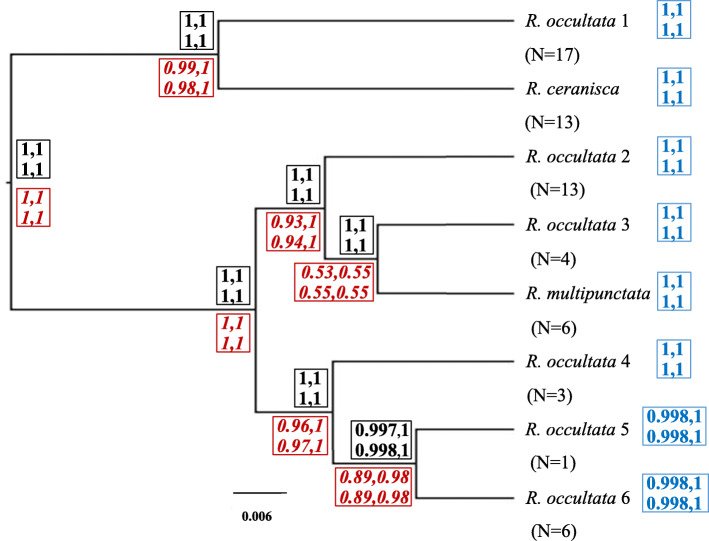
Species delimitation and topology results of BPP analyses (guided and unguided) using an 8 species model as prior on the maximum number of species. The species tree is the guide tree (derived from the mtSSU gene tree in Fig. 2a) used for the guided 8 species analyses and also the MCC (maximum clade credibility) and MAP (maximum posterior probability) topology from the unguided analyses. Values above nodes (in bold and black colour) are mean posterior probabilities (of two runs) for a species split from guided analyses. Values following terminal names (in blue colour) are mean posterior probabilities (of the two runs) of each species from the unguided analyses, accounting for phylogenetic uncertainty (i.e. not conditional on the depicted guide tree). Values below nodes (in italic and red colour) are mean posterior probability clade support values from the unguided analyses. Four probability values are given for each representing the results from the four prior variation settings in the order tau0.1theta0.1 and tau0.1theta0.01 in upper row, tau0.01theta0.1 and tau0.01theta0.01 in lower row. N indicates the number of specimens. The scale bar indicates expected number of substitutions per site

All analyses based on the 8 species guided (A10) analyses resulted in highest posterior probability to eight delimited species independent of prior combinations used (Fig. 3; Additional file 3). The clades correspond to what is here called *Rostania multipunctata*, *R. ceranisca* and six species of *“R. occultata”*. The eight species model was overwhelmingly supported over any delimitation with fewer species and ranged in posterior probability between 0.997 and 1 for both the guided and unguided analyses. The marginally lower probability occurred when using the higher prior 0.1 on theta, but this still only meant a minute posterior probability of 0.003 that *R. occultata* 5 and 6 together form one species. The unguided analyses gave the highest posterior probability support (0.425–0.536) to the species tree topology provided for the guided analyses. However, topologies with two alternative arrangements within the clade *R. multipunctata*, *R. occultata* 2 and *R. occultata* 3, received some support (0.178–0.245) under the different prior settings. Excluding the mitochondrial gene did not affect the support for the eight species delimitation model. However, the topology probability was dispersed across a larger number of alternatives in the unguided analysis, and for the prior combination theta = 0.1, tau = 0.1 a different arrangement for this clade had marginally higher probability than the guide tree (Additional file 3).

**Fig. 4:**
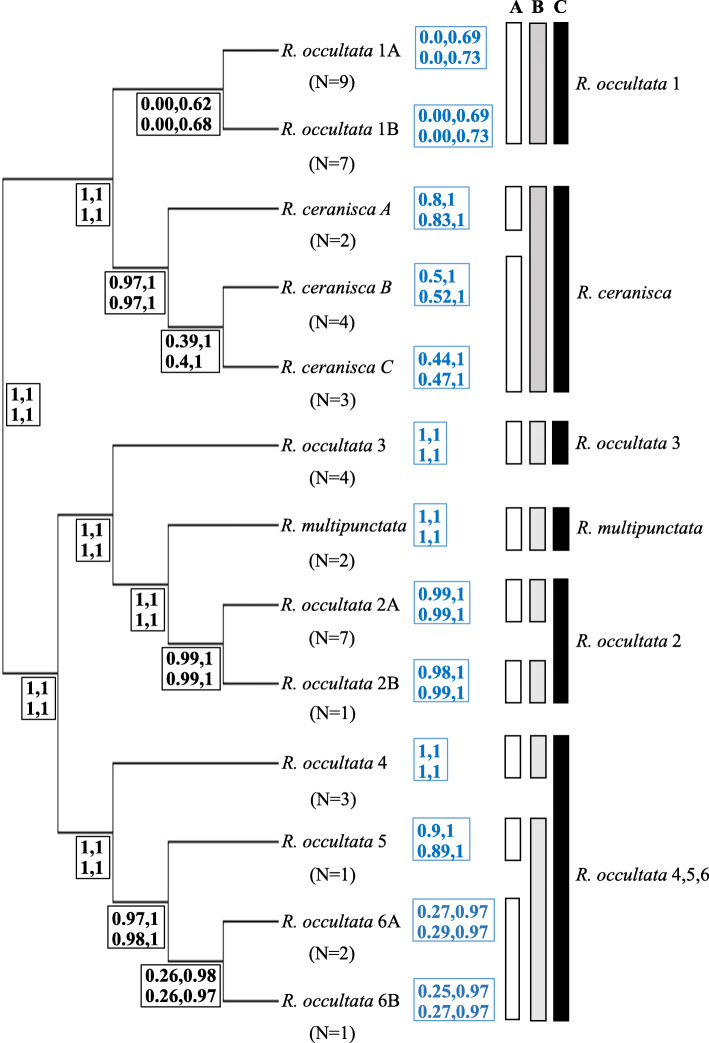
Species delimitation result of BPP analyses (guided and unguided) using a 13 species model as prior on the maximum number of species. The species tree is the guidetree (derived from the β-tub gene tree in Fig. 2b) used for the guided 13 species analyses. Node values (in bold and black colour) are mean posterior probabilities (of the two runs) for a species split from the guided analyses. Values following terminal names (in bold and blue colour) are mean posterior probabilities (of the two runs) of each species from the unguided analyses, accounting for phylogenetic uncertainty (i.e. not conditional on the depicted guide tree: see Fig. 5 for the MCC topology from the unguided analyses). Four probability values are given for each representing the results from the four prior variation settings in the order tau0.1theta0.1 and tau0.1theta0.01 in the upper row, tau0.01theta0.1 and tau0.01theta0.01 in the lower row. **a** Delimitation of 10 well-supported candidate species from the guided analyses, **b** Delimitation of 8 well-supported candidate species from the unguided analyses, **c** Delimitation of 6 conclusively supported species for which unique morphology was also found as corroborating evidence. N indicates the number of specimens

In contrast, the guided BPP analyses based on 13 species showed that some species are dependent on prior combinations used and hence 13 species are not unequivocally supported (Fig. 4; Additional file 3). The best species delimitation model in the guided analyses ranged from 10 to 13 species and was dependent on the theta prior. With theta = 0.01 the 13 species model had highest probability but < 0.95 (0.60–0.66, Additional file 3), followed by a 12 (0.31) or 11 (0.22) species model. With theta = 0.1 a model with 10 species had the highest posterior probability (0.41) followed by an 11 species model (0.28). The split for species that were not supported at > 0.95 or where such support was prior dependent were *R. occultata* 1A from *R. occultata* 1B, *R. ceranisca* B from *R. ceranisca* C and *R. occultata* 6A from *R. occultata* 6B (Fig. 4). Other splits were under all prior combinations supported at > 0.95 in posterior probability. This included support to the following ten species: *Rostania occultata* 1A + 1B, *R. ceranisca* A, *R. ceranisca* B + C, *R. multipunctata, R. occultata* 2A, *R. occultata* 2B, *R. occultata* 3, *R. occultata* 4, *R. occultata* 5 and *R. occultata* 6A + 6B, (Fig. 4). The highest support (0.015–0.02) in any analysis for a nine species model merged either *R. ceranisca* A + B + C or *R. occultata* 5 + 6A + 6B. The prior on tau had negligible effect.

The unguided 13 species analyses supported again between 10 and 13 species under the different prior settings, the latter only at theta = 0.01. At theta = 0.1 the posterior probability was similar (0.25–0.27) between the 10 and 11 species model (Additional file 3), and it was the same ten species that was supported as under the guided analysis. However, support for *R. ceranisca* A (0.80–0.83) and *R. occultata* 5 (0.89–0.90) dropped below 0.95 at theta = 0.1 in contrast to the guided analysis (Fig. 4; Additional file 3). The best nine species model that merged either of these two with *R. ceranisca* B + C and *R. occultata* 6A + 6B respectively, received slightly higher support (0.024–0.038) than in the guided analysis (Additional file 3). It is worth pointing out that even if the 13 species model have the highest posterior probability when theta = 0.01 in both guided and unguided analyses, not all 13 species had support at > 0.95.

There were some consistent differences (similar in two independent runs) between using algorithm 0 and 1 especially in the unguided 13 species analyses. For instance, at theta = 0.01 and tau = 0.01, algorithm 1 supported the 13 species model over the 12 species model in both runs with more than double in posterior support (0.7–0.71 vs 0.26–0.27) but with algorithm 0 the difference in support was reduced (0.51–0.56 vs 0.41–0.46) (Additional file 3). Although we ran the analyses with a haploid genome specified, we also tested assuming a diploid genome. This generally increased the support for the species delimitation models with a higher number of species (e.g. in unguided analysis the 13 species model had a support of 1.0 with theta = 0.01 and tau = 0.01), but did not otherwise alter the overall conclusions. This was also the case when using topology prior 0 instead of 1. At prior combination theta = 0.1, tau = 0.1, topology prior 1 resulted in most posterior support being distributed on 10 and 11 species models whereas with topology prior 0 most posterior support was distributed on 11 and 12 species models. Topology priors 2 and 3 in the unguided analyses did not result in any significant differences compared to prior 1. In no case did any variations to the analysis settings result in a species delimitation model of less than 10 species receiving highest posterior probability, or a probability > 0.1 (Additional file 3). Neither did any variations to the analysis settings result in support of < 0.95 for any of the eight delimited species (Fig. 4): *Rostania occultata* 1A + 1B (*R. occultata* 1), *R. ceranisca* A + B + C, (*R. ceranisca*), *R. multipunctata, R. occultata* 2A, *R. occultata* 2B, *R. occultata* 3, *R. occultata* 4, *R. occultata* 5 + 6A + 6B (*R. occultata* 5,6).

**Fig. 5:**
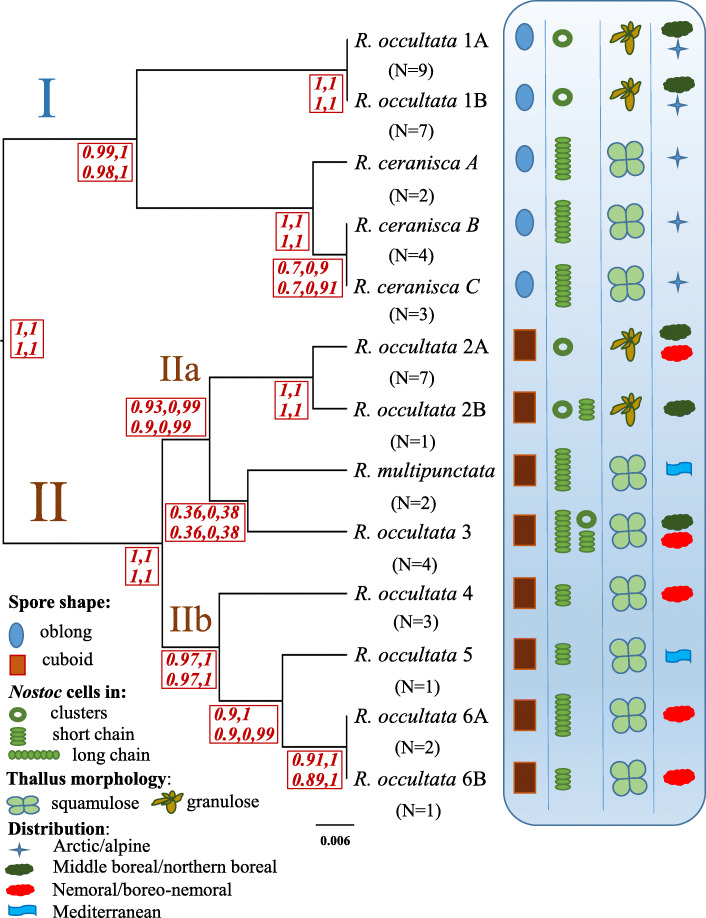
Species tree topology from the unguided BPP analyses using a 13 species model as prior on the maximum number of species. The species tree is the maximum clade credibility topology (under all four prior settings) with median node heights calculated from the tau0.1theta0.1 analysis. Node values (in red and italic) are clade support values (mean value of the two runs). Four probability values are given for each node representing the results from the four prior variation settings in the order tau0.1theta0.1 and tau0.1theta0.01 in the upper row, and tau0.01theta0.1 and tau0.01theta0.01 in the lower row. The mycobiont spore shape, photobiont morphology, lichen thallus shape, and the approximate geographical distribution based on all 63 specimens are given with symbols. The scale bar indicates expected number of substitutions per site. N indicates the number of specimens

The unguided BPP 13 species analyses (A11) provide estimation of the species tree with clade support values under the multispecies coalescent model simultaneously with the species delimitation (Figs. 4, 5). The species tree (Fig. 5) is basally divided into two clades (I and II) that are both strongly supported (0.98–1.0) under all four prior settings. *Rostania occultata* 1 (1A + 1B) and *R. ceranisc*a (A + B + C) make up clade I and are sister taxa. Clade II is composed of two subclades (IIa and IIb) that are also strongly supported (0.97–1.0) except slightly lower (0.91) for clade IIa under a high theta prior (0.1). Subclade IIa includes *Rostania occultata* 2A and *R. occultata* 2B, *R. occultata* 3 and *R. multipunctata*. *Rostania occultata* 2A and *R. occultata* 2B form together a strongly supported monophyletic group (1.0) but how *R. multipunctata* and *R. occultata* 3 is related to that clade or to each other has no resolution with even moderate support (< 0.5) as this clade is collapsed into a trichotomy in a majority rule consensus tree. The three alternative topological arrangements have almost equal level of support (Additional file 3). The resolution of the same clade varied also in the 8 species unguided analysis (Fig. 3). Subclade IIb (Fig. 5) includes *Rostania occultata* 4, *R. occultata* 5 and *R. occultata* 6 (6A + 6B) with support > 0.95. There is a moderate (0.898, theta = 0.1) to strong (0.985–0.986, theta = 0.01) support for *R. occultata* 5 and *R. occultata* 6 together forming a monophyletic group.

### Independent lines of evidence, thallus morphology and correlation with cyanobiont morphology

The species delimitation analyses indicate the presence of eight strongly supported candidate species in *Rostania* (*R. occultata* 1, *R. ceranisca*, *R. multipunctata, R. occultata* 2A, *R. occultata* 2B, *R. occultata* 3, *R. occultata* 4, *R. occultata* 5,6). We *a posteriori* examined morphology, substrate preferences and geographic distribution of the samples in order to evaluate each candidate species with independent evidence. As we will formalize the taxonomy and nomenclature problems in a separate paper that will include a detailed discussion on morphological characteristics and measurements, we do not detail these results here, but only comment on the most obvious traits.

**Fig. 6:**
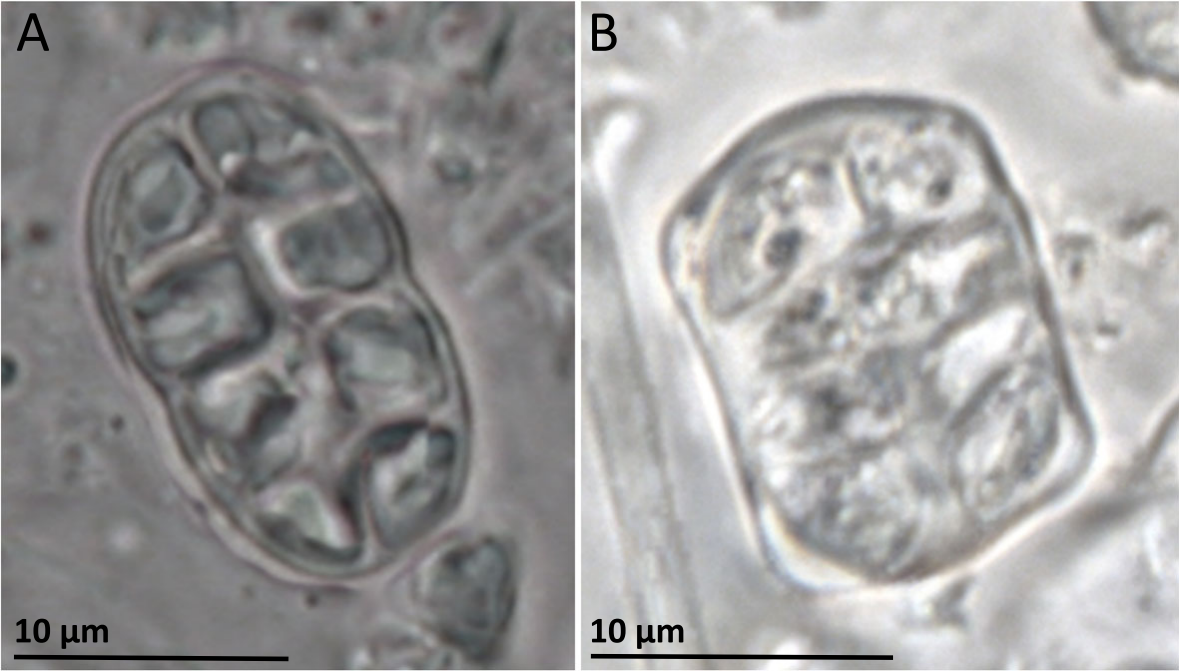
Spores in the *Rostania occultata* species complex. **a** *R. occultata* 1 (UPS-L520806), oblong to ellipsoid spore, **b** *R. occultata* 2 (S-F388749), cuboid spore

**Fig. 7:**
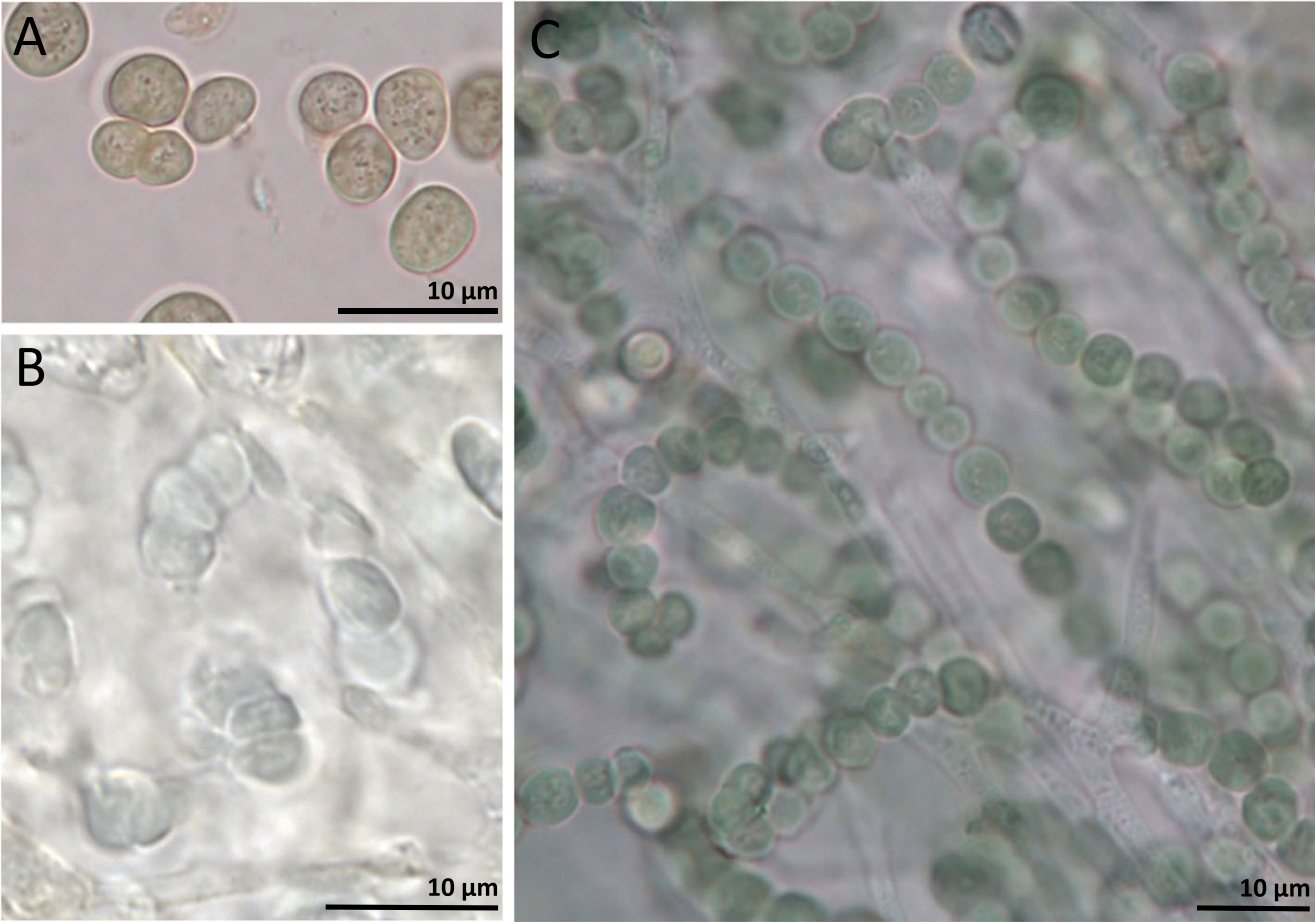
Representatives of *Nostoc* morphotypes in the *Rostania occultata* species complex. **a**) *Nostoc* cells in clusters (UPS-L834451), **b** *Nostoc* cells in short chains (S-F178903), **c** *Nostoc* cells in long chains (S-F332481)

The only arctic-alpine and terricolous species, *R. ceranisca*, is morphologically easily distinguished from all other *Rostania* species, as it has a thallus with finger-like accessory lobules (Fig. 1a). In addition *R. ceranisca* has oblong spores, something which is only shared with *R. occultata* 1 (Figs. 5 and 6a). All other species in the *R. occultata* complex have cuboid spores (Fig. 6b). *Rostania occultata* 1 is also easily characterised, as the apothecia have very pale discs (Fig. 1c). This is in contrast to all other species in *Rostania s. str.,* which have apothecia with reddish or brownish discs (Fig. 1d). *Rostania occultata* 1 is otherwise morphologically similar to *R. occultata* 2 in having a thallus composed of small granular to coralloid structures (Fig. 1c), and a *Nostoc* cyanobiont where the cells form small clusters (Fig. 7a) (except for the single specimen of *R. occultata* 2B, which has *Nostoc* cells in short chains, Fig. 5). All other species presently included in *R. occultata* have a thallus composed of minute lobes (Fig. 1d), and a *Nostoc* cyanobiont where the cells form short (Fig. 7b) or long chains (Fig. 7c). *Rostania multipunctata* forms a group with *R. occultata* 2 and *R. occultata* 3, but differs in having significantly larger lobes (up to 2.5 cm, Fig. 1b) compared with those in the *R. occultata* complex (up to ca 3 mm long, Fig. 1d). *Rostania occultata* 3 differs from *R. occultata* 4 and *R. occultata* 5,6 in anatomical details in the apothecium margin. *Rostania* occultata 3 is the only species where we have observed all three morphotypes of *Nostoc* as cyanobionts (Fig. 5). *Rostania occultata* 4 is morphologically very similar to *R. occultata* 5,6, sharing similar thallus morphology, distribution, morphotypes and shape of the spore (Fig. 5). Here, we have found small differences in *Nostoc* specificity. *Rostania occultata* 4 has *Nostoc* in short chains only (Fig. 7b) while *R. occultata* 5,6 share *Nostoc* in short and long chains (Fig. 7b, c).

Our morphological analysis further indicates a correlation between the *Nostoc* morphotypes and *Rostania* thallus morphology (Fig. 5). In *Rostania* species with a granulose thallus (Fig. 1c), *Nostoc* form clusters of cells (Fig. 7a), a correlation occurring independently in *R. occultata* 1 and *R. occultata* 2 (Fig. 5). Species of *Rostania* with a lobate thallus (Fig. 1a, b, d) share as a main cyanobiont *Nostoc* instead forming short (consisting of 3–5 cells, Fig. 7b) to long chains (Fig. 7c). This included both species with thallus composed of large lobes (*R. ceranisca* and *R. multipunctata*; Fig. 1a, b) and those with a thallus composed of small lobes (*R. occultata* 3, *R. occultata* 4 and *R. occultata* 5,6, Fig. 1d).

## Discussion

All analyses, both single locus and multi-locus species delimitation analyses, unambiguously show that *Rostania occultata* is a species complex with more species than presently recognised. However, both the number and the selection of delimited species varied to some degree between analyses (Additional Table 2).

The single-gene GMYC analyses resulted in a minimum of seven species by mitochondrial SSU (Additional Table 2; Fig. 2a), but all three nuclear genes separated a minimum of eight species. The additional eighth species is *R. occultata* 5, which is a single specimen from southern Europe. It is possible however that it either belongs to a southern population of *R. occultata* 6 and is in the early stage of speciation, or that it merely represents a genetically distinct allopatric population. The current sampling, one sample only, is insufficient to resolve this question. The GMYC method is sensitive to incomplete population sampling and migration/gene flow [46, 57, 58]. The sampling represented by one specimen of *R. occultata* 5 from a low altitude Mediterranean locality (Greece) and a number of specimens of *R. occultata* 6 from southern Scandinavia and central/western European mountain ranges, is the type of situation where GMYC may oversplit. Additional samples from geographically intermediate populations could thus change the result of the analysis [57].

The sensitivity of population sampling and migration/gene flow by GMYC is mirrored by a similar recent critique of multilocus species delimitation with the multispecies coalescent model in general and with BPP in particular [3, 59]. In short, does BPP delimit species or genetic structure between populations? A simulation study using a protracted speciation model showed BPP has a strong bias to overestimate the number of species and basically identifies genetic structure rather than true species ([3]; but see Leaché et al. [60] for an explanation given the simulation settings). The degree of bias depended on the conversion rate between incipient species and true species in the model. According to another study BPP was likewise found to oversplit, detecting two species in simulations even at high rates of gene flow between them, questioning how the delimited units really should be interpreted [59]. As originally stated for the method [43], interpretation of BPP results is most straightforward for taxa in sympatry [60]. When populations that exist in sympatry are genetically distinct there seem to be no controversy in assigning them species status [60]. In allopatry however, units delimited by BPP must be interpreted with caution, keeping in mind the growing power of BPP to detect ever-smaller population genetic divergences with increasing number of loci [3]. For allopatric populations, model selection can be complemented with heuristic criteria based on population divergence parameters (tau, theta, migration rates) estimated in a Bayesian framework from the data [3, 59]. Species delimitations from BPP should also be confronted with independent data such as morphology or ecology, and not be the sole criterion for the erection of new species [3, 6].

Results of BPP may be sensitive to prior settings, in particular if priors are set as informative distributions with a mean order of magnitude too large or too small for the data [61]. We found the outcome of BPP analyses in some instances to be sensitive to the prior on theta but not tau, in agreement with previous studies [50, 62, 63]. A prior distribution with a smaller mean on theta generally results in stronger support for more species. As we had no external information to inform the prior on theta, we took the conservative approach to not consider species splits to be well supported (> 0.95) if the support was sensitive to the prior on theta (0.1 or 0.01). For the guided analyses, this would have resulted in ten well supported species. However, it turned out that both the parameter priors and accounting for phylogenetic uncertainty influenced the support. When the topological uncertainty of the species tree was accounted for in the unguided analysis, the support for two of the ten species decreased. The support for *R. ceranisca* A as a separate species dropped from 1 to 0.80–0.83 (Fig. 4) and for *R. occultata* 5 from 1 to 0.89–0.90 under theta = 0.1 priors. It is possible that the prior delimitation model context may also have affected the outcome. When *R. occultata* 5 is tested in the 8 species BPP guided and unguided analyses context, it was supported as a distinct species under all prior settings (Fig. 3) but not when tested within the unguided 13 species context (Figs. 4-5). However the datasets were not identical in all other aspects. In the 13 species analyses 17 specimens that lacked the β-tub gene where excluded, resulting in fewer samples (see results and material and methods). *Rostania occultata* 5 and 6B were singletons in all analyses. *Rostania occultata* 6A on the other hand was represented by two south Scandinavian specimens in the 13 species analyses but with an additional three central European specimens in the 8 species analyses (Additional file 1). The increased specimen sampling for the 8 species analyses hence strengthened the hypothesis of *R. occultata* 5 as a separate species, in contrast to expectations from an artificial oversplitting-hypothesis due to incomplete sampling. However, *R. occultata* 5 is a singleton and therefore, for the time being, we consider this single sample as part of the same species as *R. occultata* 6 (*R. occultata* 5,6).

The selection of loci could also have affected the species delimitation results. We analysed multiple gene fragments separately with GMYC and combined under the multispecies coalescent model. Zakeri et al. [64] pointed out that a strong phylogenetic signal from mtSSU may have dominated their analysis of the concatenated genes. We compared analyses with all data with a reduced nuclear dataset excluding the mitochondrial locus but this had marginal effect on the result (Additional file 3). Our results are not dominated or overly influenced by the mitochondrial marker.

### Taxonomical consequences

Conclusive correlation between morphological/ecological characters and genetic delimitation could be found for six of the eight candidate species, namely for *R. ceranisca*, *R. multipunctata*, *R. occultata* 1, *R. occultata* 2, *R. occultata* 3 and *R. occultata* 4,5,6. For two of the eight species, *Rostania occultata* 2B and *Rostania occultata* 4, independent lines of evidence are still insufficient for recognition at species level. All genetic analyses suggest that *Rostania occultata* 2B represents a distinct species (Figs. 4, 5). This is further supported by thallus morphology in that *R. occultata* 2B has a matt thallus surface compared to shiny surface in all samples of *R. occultata* 2A. Additionally, *Nostoc* cells in *R. occultata* 2A form small clusters while *R. occultata* 2B has *Nostoc* both in clusters and in short chains. However, *Rostania occultata* 2B is based on a single sterile sample and therefore it is not possible to assess the validity of these characteristics sufficiently and conclusions have to await the discovery and examination of additional specimens, including fertile thalli. *Rostania occultata* 4 is a more convincing case in that the molecular delimitation is based on a larger number of samples. Nonetheless, we have so far not been able to find any morphological or ecological characters to distinguish this putative cryptic species from *R. occultata* 5,6. Hence, under the unified species concept we find corroborating independent lines of evidence to support six species in *Rostania*, while two additional candidate species require further investigation and sampling. Apart from *R. ceranisca* and *R. multipunctata*, the other four species belong to the non-monophyletic *R. occultata* sensu *lato* complex. Neither of Degelius’ [28] two varieties of *“Collema” occultatum* correspond to monophyletic species. *Rostania occultata* var*. occultata* includes both *R. occultata* 1 and *R. occultata* 2 whereas *R. occultata* var. *populina* includes *R. occultata* 3 as well as *R. occultata* 4,5,6 (Figs. 3, 4 and 5).

The basal division in the species tree (Fig. 5) is supported by the spore shape. Species in clade I all have oblong to ellipsoid spores (Fig. 6a) in contrast to the species in clade II which all have cuboid spores (Fig. 6b). Some geographical structuring is also clear as species in clade I (Fig. 5) have an arctic-alpine (*R. ceranisca*) or northern boreal (*R. occultata* 1) distribution. With the exception of *R. occultata* 2 which has a northern distribution very similar to *R. occultata* 1 (Additional file 1)*,* species in clade II have a more southern distribution ranging from the middle boreal to the nemoral zone, sometimes with occurrences as far south as in the Mediterranean region (*R. multipunctata* and *R. occultata* 4,5,6).

## Conclusions

We used multilocus sequence data, likelihood and Bayesian species delimitation frameworks to test species limits within *Rostania*. We conclude that *Rostania* includes a minimum of six species which will be formally treated in a taxonomical paper: *R. ceranisca*, *R. multipunctata*, *R. occultata* 1, *R. occultata* 2, *R. occultata* 3, and *R. occultata* 4,5,6. *Rostania occultata* 4 and *R. occultata* 2B were also unequivocally supported as distinct species based on the molecular analyses but the latter is based on a single sterile specimen and for the former we have yet to identify non-molecular evidence. There is a possibility that additional candidate species represent separately evolving units as well (*R. ceranisca* A and *R. occultata* 5), but neither the morphology nor the genetic support across analyses were conclusive enough to suggest that they should be recognised as species. Three distinct *Nostoc* morphotypes occur in *Rostania*, and there is substantial correlation between these morphotypes and *Rostania* thallus morphology.

## Materials and methods

### Taxon sampling

We sampled 63 specimens, representing as much of the morphological, geographical, and ecological diversity of *Rostania occultata s. lat.* as possible from Scandinavia, and including some extra-Scandinavian material for comparison. This includes all species within *Rostania s. str.* as delimited by our previous study [29] and the two varieties accepted in *R. occultata* [28, 30]. Most of the collections result from our own recent fieldwork, and are complemented by recent herbarium samples from areas we have not covered. The sequences used are summarized in Additional file 1. Our new collections are deposited in UPS and S, and additional material from the herbaria AMNH, BG, GZU, UPS and S were also used. Herbarium acronyms follow Index Herbariorum [65].

We studied morphological and anatomical characters under light microscope and dissecting microscope. We used transversal hand-cut sections of apothecia and lobes in water, to observe internal and microscopic characteristics.

### DNA extraction, amplification, sequencing and sequence alignment

Two apothecia with surrounding thalline parts, or, in the case of sterile samples, a thallus fragment were selected for extraction. We extracted total DNA using the Plant DNA Mini Kit (Qiagen, Hilden, Germany) following the manufacturer’s instructions except in order to increase the concentration of DNA we used half the amount of Elution buffer in the last step. We amplified the small subunit of the mitochondrial rDNA (mtSSU), the two protein-coding genes β-tub and DNA replication licensing factor mini-chromosome maintenance complex component 7 (MCM7), and partial RPB2 protein-coding gene divided into two parts − 5-7 (RPB2 5–7) and 7–11 (RPB2 7–11).

The mtSSU rDNA was amplified using the primers mrSSU1 and mrSSU3R [66] and for sequencing we additionally used the internal primers mrSSU2 and mrSSU2R [66]. The protein coding β-tub was amplified and sequenced using the primers Bt3-LM5 and Bt10-LM3 [67], BetaCollF and BetaCollR [25] and the newly designed internal primers for Collemataceae **BetaColl-intF2** (TGG CAT GGG CAC TTT RTT GAT YTC) and **BetaColl-intR** (ATC GGA ATT CTC CAC CAA YTG ATG). The PCR primers were used in the following combinations: Bt3-LM5/Bt10-LM3, BetaCollF/BetaCollR, Bt3-LM5/BetaCollR, BetaCollF/Bt10-LM3 (the best working combination), BetaCollF/BetaColl-intR and BetaColl-intF2/Bt10LM3. The MCM7 gene was amplified and sequenced using the primers Mcm7-709for and Mcm7-1348rev [68]. The locus RPB2 was amplified as two separate parts using the primers fRPB2-5F, fRPB2-7cF, fRPB2-7cR and fRPB2-11aR [69] and also here, the PCR primers were used as sequencing primers. PCR amplifications were performed using Illustra™ Hot Start PCR beads, according to manufacturer’s instructions. PCR-reactions were performed using the same settings as in previous studies [25, 29, 70]. We assembled and edited DNA sequences using Geneious version R8 (http://www.geneious.com) [71].

Alignments were constructed separately for each gene with the “ClustalW/Multiple alignment” option and subsequent manual adjustments. The two parts of RPB2 – RPB2 5–7 and RPB2 7–11 - were treated as one marker, concatenated without overlapping but keeping the reading frame using AliView 1.09 [72]. The trimming of the 5’ and 3’ ends was required due to the poor sequences quality.

### Species delimitation analyses using GMYC

The GMYC method can be applied in cases with no prior knowledge of putative species limits. An advantage of GMYC is that the likelihood framework allows for statistical inference and hypothesis testing across the entire sampled clade. The GMYC analyses [40] were performed in the R statistical package [73] utilizing the ape, gee, MASS, paran and splits packages [74, 75]. The GMYC method takes as input an ultrametric gene tree, which we derived from a Bayesian analysis using a clock model in BEAST v. 2.5.2 [76]. We calculated the maximum clade credibility tree with mean node heights in Tree Annotator from the sampled mtSSU, β-tub, MCM7 and RPB2 5–11 gene trees separately.

For the BEAST analyses we used the partition scheme and the optimal model of nucleotide substitution as suggested by PartitionFinder2 [77–79]. PartitionFinder settings were: branch lengths linked, data blocks as one for mtSSU or divided according to each codon position for the protein coding genes (β-tub, MCM7, RPB2 5–11), the greedy search scheme and only models available in BEAST. The selected partition and substitution model schemes are provided in Additional file 4. In BEAST, we ran all single marker analyses as two separate runs independently for 10 million generations, sampled every 1000 generations. We used Tracer [80] to examine the convergence across runs and ESS values of sampled parameters (minimum > 100, optimal > 200). After 200.000 trees from each run were discarded as a burn-in, the remaining samples from both runs were pooled and the maximum clade credibility species tree was calculated using the mean node heights. We selected a strict or relaxed (lognormal) clock model on branch lengths for the BEAST analyses (Additional file 4) following a molecular clock test [81] based on the harmonic mean estimate of marginal likelihood in MrBayes 3.2 [82].

The GMYC method assumes that independent evolution leads to the appearance of distinct genetic clusters, separated by long branches, in gene trees derived from datasets with multiple species and multiple individuals per species [83, 84]. It delimits such clusters by optimizing a threshold defining the transitions between inter and intra-specific processes. GMYC calculates the likelihood of a mixed Yule-coalescence model applied with a single threshold across the tree at every node. At each threshold, deeper nodes are calculated as speciation events according to a Yule model [85] whereas each group of younger nodes is calculated separately according to the coalescent process model [86]. The maximum likelihood solution generally identifies the point of increased diversification rate in a lineage-through-time plot of trees with multiple individuals per species for multiple species. This node serves as a species delimitation point under the assumption of species monophyly and not permitting any speciation event to be younger than the deepest coalescence.

The maximum likelihood of the GMYC model was tested with a likelihood ratio test against a null model treating the entire tree as a single coalescent (i.e. against a one-species model).

### Bayesian species delimitation analyses using BPP

Bayesian Phylogenetics and Phylogeography (BPP) is a software for Bayesian estimation of species limits using multi-locus data under the multispecies coalescence model (MSC) [87–89]. It uses reversible-jump Markov Chain Monte Carlo (rjMCMC) to sample different species delimitation models with either a fixed species tree (the “guide tree”) or with the species tree co-estimated simultaneously [43, 90]. The analysis using a guide tree (guided analyses) infers the species delimitation using a user-defined tree topology and specimen assignment to species as input. The reversible-jump MCMC algorithm is used to jump between species delimitation models that are compatible with the guide tree [90, 91]. Collapsed versions of the guide tree representing fewer species are evaluated, but not versions where the initial assignment is split further. Non-guided analyses (unguided analyses) estimate both species delimitation and species tree topology simultaneously. As in guided analyses the assignment of individuals into populations is fixed and only models with equal or fewer number of species as in the initial assignment are evaluated. A prior on a maximum species division is therefore required for the analysis.

We ran both guided (A10) and unguided (A11) species delimitation analyses in BPP [42, 43, 90]. We first defined an 8-species guide tree for the 8 species guided analyses as a prior following morphological evidence and the minimum delimitation from the single-locus GMYC-analyses. The exception was the mitochondrial gene, which did not separate single specimen AL264 in contrast to the unanimous evidence in all three nuclear markers (Fig. 2A-D). By separating AL264 in the guide tree, the hypothesis of merging the terminal with the cluster suggested by the mitochondrial marker will be tested. We used the mtSSU gene tree topology as the guide tree, but in the unguided (A11) analyses this is only used as starting tree with alternative topologies evaluated (Fig. 2a). As the 8-species model was very strongly supported over all models with fewer species in all analyses (Additional file 3) we also defined a 13-species guide tree for 13 species guided analyses. The latter followed the GMYC result of the β-tub gene which delimited a minimum of 11 species (Fig. 2b), subdivision of *R. occultata* 1 (to 1A and 1B) and *R. ceranisca* to (A, B and C) on top of the other eight species. Additionally, the two singletons *R. occultata* 2B (AL315) and *R. occultata* 6B (MW87) that were delimited as separate units in at least one of the other genes (Fig. 2c) were separated in the 13 species analyses. All specimens which were not sequenced for β-tub and therefore not possible to assign to the 13 hypothetical species were excluded from these analyses, resulting in a combined matrix of 46 terminals. All samples and their presence in 8 or 13 hypothetical species analyses are summarised in Additional file 1. We investigated the effect of orders of magnitude variations to the theta and tau priors as these are known to potentially influence the results [61]. The theta parameter is equal to 4Neu where Ne is the effective population size and u is the mutation rate per site per generation. There is a separate theta parameter for each included species in the model (as long as the species has more than one representative in at least one locus) and one for each of the ancestral nodes. The prior on theta is the same for all theta parameters in the model. Tau is the divergence time at each branching point in the species tree and there are s-1 tau parameters where s is the number of species. The prior on tau is only for tau_root_ and priors for the remaining tau parameters are generated from a uniform Dirichlet distribution [43]. We set a low value (2) to the shape parameter of an inverse gamma distribution to form diffuse priors on theta and tau reflecting our lack of prior knowledge. Genetic distance calculations and preliminary runs were used to test what ranges for theta and tau are reasonable for the data [42]. We concluded that reasonable values for both parameters are in the range between 0.1 and 0.01, and following this we set the inverse gamma rate parameter so that, given a shape parameter of 2, the mean of the distribution was 0.1 or 0.01. To encompass the uncertainty of the true value, we then tested the effect of four combinations of rate parameters to the inverse gamma distribution as priors to tau and theta (in parentheses mean of the distribution): i) tau (0.1), theta (0.1), ii) tau (0.1), theta (0.01), iii) tau (0.01), theta (0.1), and iv) tau (0.01), theta (0.01). We used inverse gamma distributions which is default in BPP 3.4 (in contrast to normal gamma distributions in earlier versions) since it allows theta to be integrated out analytically so that it is not a state of the Markov chain and is not estimated [92]. The reduction in state space is supposed to improve mixing between trees and delimitation models [92]. We used a heredity scalar for effective population size to set the mitochondrial marker to either one fourth (main analyses) or one half (sensitivity analyses) of the nuclear markers, assuming haploidity for nuclear markers. Loci were allowed variable mutation rates with a Dirichlet distribution following the random-rates model [93] where the average rate across loci is fixed to 1. At two of the prior combinations (tau (0.01), theta (0.01) and tau (0.1), theta (0.1)) we tested that 1) estimation of theta did not affect the other model parameters and the species delimitations, 2) algorithm 0 gave the same result as algorithm 1 [43], 3) excluding the mitochondrial locus and only running nuclear genes gave similar results as all combined, 4) for comparison, assuming a diploid genome and adjusting the relative effective population size between nuclear and mitochondrial loci did not alter the result, and finally 5) we evaluated the effect of one (guided) or three (unguided) alternative topology priors described by [42]. In all main analyses the default prior 1 was used which assigns equal probability to all rooted trees with different species delimitation models. With the topology fixed (guided analyses-A10) there were for instance 21 such models with the 8 hypothetical species guide tree used. Under alternative prior 0 equal probability is put on all labelled histories, giving balanced trees higher probability than unbalanced. In the unguided (A11) analyses, there is also the option of assigning equal probability to the number of species, divided either uniformly (prior 3) or (prior 2) in proportion to the compatible number of labelled histories [42].

Fine-tuning parameters for the proposals in the MCMC algorithm were left as default. For the analyses with the 8 species (guided and unguided) we ran 10,000 generations as pre-burnin followed by 200,000 generations, sampled every second generation, of the Markov chain. For the analyses with the 13-species (guided and unguided), 200,000 generations were not enough why these were run for one million generations (preceded by 100,000 generations as pre-burnin), sampled every fifth generation. Each analysis was run twice to check convergence of results. Mean posterior probability for delimitations was calculated from the two runs. The maximum clade credibility topology was calculated by Treeannotator for the unguided analyses after tree samples from the two independent runs had been merged. We consider a posterior probability of > 0.95 as strongly supported.

## Supplementary information

**Additional file 1.** Specimens and sequences utilized in this study including newly produced sequences (in bold), from reference [20] (21 sequences) and from reference [17] (1 sequence). Each specimen has a unique DNA extraction code. The analyses included four genes, mtSSU = small subunit of the mitochondrial rDNA, MCM7 = replication licensing factor mini-chromosome maintenance complex component 7, β-tub = β-tubulin gene. In addition, analysis of RPB2 included two parts of the gene - RPB2 5–7 = RPB2 protein-coding gene part 5–7 and RPB2 7–11 = RPB2 protein-coding gene part 7–11.

**Additional file 2 **Summary of the results of the GMYC analyses using each individual gene separately. Asterisks indicate significance of the likelihood ratio (LR) test (*p-value < 0.05, **p-value < 0.01 and ***p-value < 0.001- highly significant).

**Additional file 3.** A-D Results of 16 species delimitation analyses (× 2 runs) with BPP with (A10) and without (A11) guide tree, with 8 or 13 as the maximum number of species and under four prior combinations of theta and tau. In addition, with two prior combinations of theta and tau, the sensitivity of the results to additional analysis assumptions and algorithms settings were evaluated. Values in cells are posterior probabilities.

**Additional file 4.** Substitution model parameters and partitions according to the best scheme calculated in PartitionFinder. The models for each codon-position partition of the protein coding genes were estimated separately. Appropriate molecular clocks were calculated in MrBayes for all codon-positions in each gene combined.

## Data Availability

The gene data set supporting the results of this article is available in Genbank (http://www.ncbi.nlm.nih.gov/genbank/), and accession numbers are found in Additional file 1. The aligned matrices of the *Rostania* analyses (mtSSU, β-tub, MCM7, RPB2 5–11 datasets,) are available at TREEBASE under URL:http://purl.org/phylo/treebase/phylows/study/TB2:S26825.

## Abbreviations

A10: Guided analyses (topology is defined)
A11: Unguided analyses (topology is not defined)
BPP: Bayesian species delimitation analyses
GMYC: General Mixed Yule Coalescence model
MCM7: Replication licensing factor mini-chromosome maintenance complex component 7
mtSSU: Small subunit of the mitochondrial rDNA
β-tub: Protein-coding gene β-tubulin
RPB2: Second-Largest Subunit of RNA Polymerase II
