# Supplementary material for: Species delimitation in the cyanolichen genus Rostania
Source: BMC Evol Biol. 2020 Sep 10;20:115. doi: 10.1186/s12862-020-01681-w (PMC7488055; doi:10.1186/s12862-020-01681-w)
Supplement: Supplementary file 2 — Additional file 2 Summary of the results of the GMYC analyses using each individual gene separately. Asterisks indicate significance of the likelihood ratio (LR) test (*p-value < 0.05, **p-value < 0.01 and ***p-value < 0.001- highly significant). [file 12862_2020_1681_MOESM2_ESM.docx]

|  | **Genetic markers** | | | |
| --- | --- | --- | --- | --- |
| **Single threshold** | **mtSSU** | **β-tub** | **MCM7** | **RPB2 5-11** |
| Likelihood of the null model (coalescent model) | 528,833 | 282,575 | 171,3953 | 382,5911 |
| Likelihood of the GMYC model | 543,079 | 290,9576 | 176,803 | 386,5024 |
| Likelihood ratio | 28,492 | 16,765 | 10,815 | 7,823 |
| Result of LR test (p-value) | 6,502e-07*** | 0,00023*** | 0,0045*** | 0,02001* |
| Number of delimited species at ML solution (confidence interval) | 7(7-20) | 11(11-13) | 10(8-16) | 8(8-24) |
| Threshold time | 0,002284 | -0,00921 | -0,01361 | -0,002484 |
